# Backers Beware: Characteristics and Detection of Fraudulent Crowdfunding Campaigns

**DOI:** 10.3390/s22197677

**Published:** 2022-10-10

**Authors:** SeungHun Lee, Wafa Shafqat, Hyun-chul Kim

**Affiliations:** 1 Department of Software, Sangmyung University, Cheonan 31066, Korea; 2 Language Technology Research Group, University of Hamburg, 22527 Hamburg, Germany

**Keywords:** crowdfunding, deception detection, scam, linguistic cues, natural language processing, feature selection

## Abstract

Crowdfunding has seen an enormous rise, becoming a new alternative funding source for emerging companies or new startups in recent years. As crowdfunding prevails, it is also under substantial risk of the occurrence of fraud. Though a growing number of articles indicate that crowdfunding scams are a new imminent threat to investors, little is known about them primarily due to the lack of measurement data collected from real scam cases. This paper fills the gap by collecting, labeling, and analyzing publicly available data of a hundred fraudulent campaigns on a crowdfunding platform. In order to find and understand distinguishing characteristics of crowdfunding scams, we propose to use a broad range of traits including project-based traits, project creator-based ones, and content-based ones such as linguistic cues and Named Entity Recognition features, etc. We then propose to use the feature selection method called Forward Stepwise Logistic Regression, through which 17 key discriminating features (including six original and hitherto unused ones) of scam campaigns are discovered. Based on the selected 17 key features, we present and discuss our findings and insights on distinguishing characteristics of crowdfunding scams, and build our scam detection model with 87.3% accuracy. We also explore the feasibility of early scam detection, building a model with 70.2% of classification accuracy right at the time of project launch. We discuss what features from which sections are more helpful for early scam detection on day 0 and thereafter.

## 1. Introduction

Crowdfunding has emerged as a lucrative alternative in acquiring investments for new startups that have faced the daunting challenges of financing over the last decade. Venture capital (VC) being in a perilous state, crowdfunding has managed to become an unwavering source of support for individuals, small businesses, startups, and industries by soliciting huge amounts from a large number of people. With $5.9 billion raised in 2021 and $6.5 billion raised in 2022 [1], the global crowdfunding industry is growing steadily every year, while the VC industry manages to invest an average of $30 billion each year [2].

The tremendous upswing and popularity of crowdfunding are associated with the convenience it provides to the concerned parties in terms of soliciting money. Crowdfunding mainly follows four types of models [3]. First, some follow the patronage model, where funders expect no direct returns for their pledges, acting as a philanthropist. Second is the lending model, where funds are provided as a loan, with some rate of return expected on them. The third approach treats funders as investors, where they are given equity stakes or similar considerations for their investments. Finally, the reward-based model, where investors are expected to receive some rewards for their investments. In this study, we focus on the reward-based crowdfunding model, the most prevalent one among the four types of models. As of May 2022, Kickstarter being the largest reward-based crowdfunding site, has raised more than $6.6 billion on 560 K projects launched [4].

Despite all the progress and fame, crowdfunding is confronting some serious challenges as well. As crowdfunding prevails, it is also under substantial risk of the occurrence of fraud [5]. The ease of exemplifying the idea, convenience in usage, flexibility in requirements, and lack of legal resources for the investors [6], have forged a platform for fraudsters to thrive. In reward-based crowdfunding, funds are raised without the creator’s legitimate testimony of commitment to delivering the promised rewards on time. For the swindlers, this often causes an opportunity to steal the money. As a consequence of that, there is always the possibility that deceivers may abuse the system and the trust of investors.

**Figure 1:**
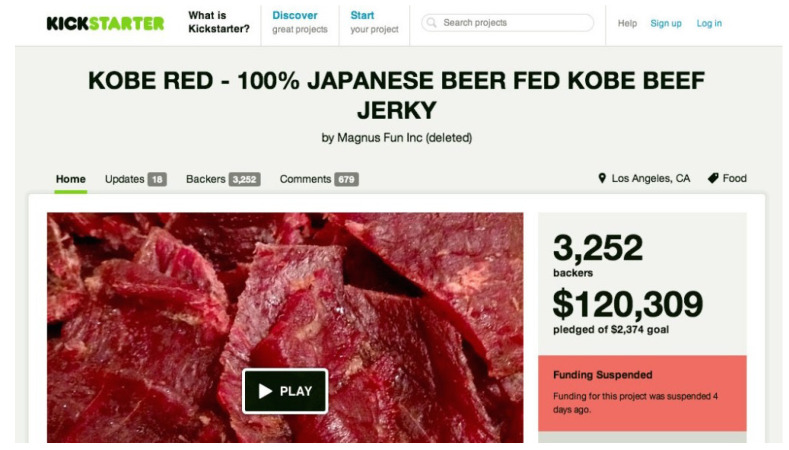
A well-known crowdfunding scam.

One well-known case of attempted crowdfunding fraud [6] is “Kobe red beef jerky” on Kickstarter shown in Figure 1, a project by Magnus Fun Inc., who claimed to provide fresh Kobe beef-based jerky from Japan and posted fake user experiences showing they loved the taste. It almost enabled a $120,309 heist, nearly 50 times the original financing goal of the campaign, from 3252 backers (i.e., investors) in just less than 1 month. Fortunately, Kickstarter pulled the plug on this fraud at the last minute of the fundraising period, as a documentary film project “Kickstarted” had publicly raised concerns and suspicions on the project’s authenticity, in an in-depth Reddit post [7,8].

According to Suspicious Activity Reports of the US Financial Crimes Enforcement Network (FinCEN) [9], a bureau of the US Department of the Treasury, the number of suspicious filed cases with crowdfunding increased by 171% between 2013 and 2015 [10]. Moreover, a sudden burst of blistering articles on crowdfunding frauds and incriminatory discussions on different sites such as reddit.com, kickscammed.com, and the Facebook group called “Kickscammed: Crowdfunding projects that never delivered”, etc., show a clear indication of anxiety and disruption in the victims and a signal that the general public needs to be protected from the inevitable incursion of the deceivers. This upsurge of alleged frauds has also caused some legal actions on the federal and state level. In 2015, the Federal Trade Commission (FTC) took the first-ever legal enforcement action against a crowdfunded project called *The Doom That Came To Atlantic City!*, showing that the FTC is willing to protect consumers taking advantage of this new and emerging financial technology [11].

The basis for the regulations and legal actions is clear: in order for crowdfunding models to survive and proliferate as an alternative, viable and lasting means of funding for emerging companies, fraud has to be limited, i.e., unsuspecting contributors, donors, and investors must be protected [12]. However, despite the fact that crowdfunding scams are a new imminent threat to investors [13], progress toward understanding them has been limited by the lack of measurement data collected from a good amount of real scam cases. This paper fills this gap by collecting and analyzing a hundred crowdfunding scam campaigns using a wide variety of traits and feature selection processes. We highlight the key contributions from our study:

a. We collect and analyze a hundred crowdfunding scam campaigns from one of the most popular crowdfunding sites, Kickstarter.com, using a wide variety of traits extracted from almost all the information and contents available on the platform; (i) generic information of campaigns such as the number of backers, updates, or comments, funding goal, etc., (ii) both profile and behavior information of campaign creators such as the availability of a link to a Facebook ID, external web pages, or an email address, the number of created or backed projects before, etc., and (iii) campaign content-based information like linguistic cues, inclusion or number of videos and images, Named Entity Recognition [14] features (e.g., names of people, locations, and organizations), etc., extracted from all the Campaign, Updates, and Comments sections available on the platform for fundraisers to pitch and communicate with backers.

b. In order to find and understand the distinguishing characteristics of scam campaigns, we propose to use the feature selection method called Forward Stepwise Logistic Regression [15]. As a result, we successfully reduce the size of the feature space from 157 to 17, with which our model classifies scams and non-scams with 87.3% accuracy. We found that features extracted from the Comments (6 out of 17) section are most helpful in detecting scams, closely followed by those from the campaign creator’s information (4 out of 17) and the Updates section (5), then by the Campaign section (2).

c. Based on the selected 17 key features, we provide our findings and insights on distinguishing characteristics of scam projects and their creators, interpreting and discussing the findings in relation to previous research. In particular, the following six features out of those 17 are our own original findings: (i) the number of images and (ii) email contacts included in the Campaign and Updates sections, more use of (iii) (modal) verbs (in scams), (iv) sentences (in non-scams), and (v) present tense verbs (scams in Comments), and (vi) past tense verbs (non-scams in Updates), etc. We also found and discussed that scammers write or behave in a different or even the opposite way across Campaign, Updates, and Comments sections, particularly in terms of pronoun usage and the number of images contained.

d. We also explore the feasibility of early scam detection, achieving 70.2% of classification accuracy using only six features available right at the time of project launch. We found that features from the creators’ information and Campaign section are helpful for 0-day scam detection (with 70.2% accuracy), and the performance increases further from day one, as more information from Updates and Comments sections becomes available later on.

The rest of this paper is organized as follows: After reviewing related work in Section 2, we describe our dataset and methodology in Section 3. Section 4 presents and discusses the results of key feature extraction and our scam classification model. Section 5 concludes the paper.

## 2. Related Work

### 2.1. Crowdfunding

Most of the work on crowdfunding has focused on predicting whether a project will successfully be funded or not. Mollick found that project static features (e.g., existence of video, spelling check, and number of updates) and social features (e.g., a creator’s number of Facebook friends), are strongly related to the success of a project [16]. Another study of his found that around 9% of all funded projects failed to deliver rewards [17]. Greenberg et al. discovered that at the time of launch, success or failure of a project can be predicted with 68% accuracy by using SVM [18]. It has been found that features like having quality video [16], quality and consistent progress updates and comments [18,19,20,21,22], creator’s backing history [23], trust relationship with backers [24], creator’s personality traits [25,26], play an essential role in leading to successful funding. Mitra et al. [27] showed that language or certain phrases used by project creators have an impact in driving the crowd to invest in a project. They certainly found specific phrases that are powerful predictors of success, and these influencing phrases are mainly related to: (1) social identity, (2) reciprocity, (3) scarcity, (4) social proof, (5) liking, and (6) authority. In another study the importance of text in debt-based crowdfunding has been highlighted. Text, in particular, contains hidden relevant features; thus, backers indeed consider the readability and length of textual information, when investing [28].

In a comprehensive economic aspect, crowdfunding being a geographical phenomenon, shows its dependence on social networks [29], as backing practices are affected by the social circles and underlying project quality [16,24,30]. Social features (e.g., related tweets or retweets to a campaign) combined with generic project features (e.g., number of backers, etc.) give higher accuracy in success prediction [31]. Lynn et al. found that strangers in crowdfunding communities from twitter play a direct role in disseminating information, investing to platforms [32]. Existing work has also focused on identifying types of investors and influential investors. Kim [33] identified two types of key investors (product and market experts) who can influence other investors, and Mollick [34] found that crowd sagacity looks to be equivalent to that of experts when it comes to deciding to fund a project. Mostly failure on Kickstarter is due to the incompetence of the creator to find potential investors [35]. Novelty of project idea, rewards and the motivation to help the community can greatly attract the right investors [36].

### 2.2. Deception, Fraud and Linguistic Cues

Deception is very common when it comes to online networks. Online social networks are mainly exposed to deceptive and fraudulent activities. Deception involves the manipulation of language and careful construction of messages or stories that appear truthful to others. There are many studies focusing on identifying the characteristics of liars, fraudsters, and deceivers by analyzing frauds in financial statements, deceiving emails, fake profiles, and deceptive conversations on dating sites, using different techniques [37,38,39,40]. It has been widely assumed by those deception models that deceivers and deception leave their footprints [41]. Text content manipulation, as we often see falsifying information on social media, is one of the most common, easy, and low cost ways to deceive others. It also has a higher probability of success due to factors like lack of resources, methodologies or efforts for verification, explanation and accountability.

The linguistic approach to deception infers that unconscious formulation of certain word types can reflect the sentiments and cognition experienced by con artists, as the choice of words in daily communication can reveal different social and psychological aspects of people [42]. Therefore, linguistic analysis has been used to detect fraud or liars, e.g., identification of deceptive profiles [39], financial fraudulent statements [43] and deceptive emails in organizations [40]. Linguistic cues were used in text-based computer mediated systems by Zhou et al. [44] and were proven very useful in detecting deception. Linguistic cues such as (1) word count, (2) pronouns, (3) expression words, and (4) exclusive words which turned out to be associated with deception [38,41]. Deceivers face a constant struggle in writing due to their lack of familiarity with what they are explaining; and to avoid conflicts with their own statements, therefore, they provide fewer details [41,45,46].

### 2.3. Detecting Fraudulent Crowdfunding Projects

As one of the earliest steps towards an empirically grounded understanding of crowdfunding scams, our earlier work [47] explored the feasibility of detecting fraudulent crowdfunding projects using linguistic features, where we showed that scammers deliberately try to deceive people by providing less information as well as writing more carefully and less informally. Gao et al. found that on an online debt crowdfunding (i.e., peer-to-peer lending) platform, a higher rate of deception cues in a loan application, such as more spelling or grammatical errors and less objective, spatial, and temporal information, is often associated with a higher likelihood of default [48]. Siering et al. showed that linguistic and content-based cues using the Bag-of-Words representation are helpful in detecting fraudulent crowdfunding projects, achieving up to 79.7% accuracy [49]. Cumming et al. [50] discovered that campaign description details (from Campaign section), campaign creators’ background and social media affinity, and campaign characteristics like funding duration are significantly related to the likelihood of detecting fraudulent crowdfunding projects.

Our model is (i) built on more varieties of traits including all the generic project-based and creator-based features, in addition to original and hitherto unused linguistic cues (e.g., Quantity: nouns, clauses, phrases, etc. Relativity: Time, past, present, and future tense verbs, etc.) and Named Entity Recognition features (e.g., names of people, locations, and organizations), collected from not only the Campaign but also the Updates and Comments sections, and then (ii) further strengthened by the feature selection process through which we obtained the 17 most distinguishing characteristics (out of 157 in total) of scam campaigns, 6 of which were our own original findings. As a result, (iii) our work achieves 87.3% classification accuracy, (iv) providing several original insights and interpretations on distinguishing characteristics of scam projects and creators. (v) We also explore the feasibility of early scam detection, showing specifically what features from which sections are more helpful, since the time of project launch.

## 3. Methodology

This section describes our methodology, including the dataset, proposed set of features to use, and performance metrics.

### 3.1. Dataset

**Figure 2:**
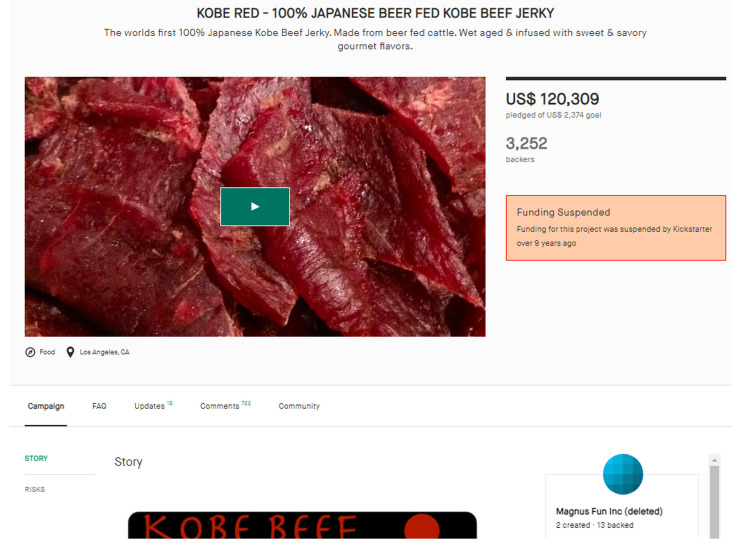
An example screenshot of a crowdfunding project.

Our dataset consists of publicly available data collected from Kickstarter. Figure 2 shows an example of a Kickstarter project. As shown in Figure 2, a crowdfunding project typically has (1) a Campaign section where the project creator introduces and describes the project idea with the help of images or videos, (2) an Updates section where the project creator keeps the backers updated with the project progress, (3) a Comments section where both backers and creator can freely leave their comments, and (4) a Community section that shows where backers come from, the top 10 cities and countries, and the number of new backers (i.e., backers that have never backed a project on Kickstarter before) and returning backers (i.e., backers had backed a project on Kickstarter before). As there is no publicly available collection of crowdfunding scam projects (i.e., ground truth), we first collected 300 campaigns accused of being scams at public forums such as Kickscammed.com, Reddit.com, and the Facebook page (Crowdfunding Projects that Never Delivered), etc., along with the disputed details or claims. These campaigns, in total, have successfully raised $11.5 million from 175,260 backers. Then, we have manually and thoroughly scrutinized all the comments and updates left on every single project for at least one year since the time of launch (up to 5 years, for the oldest projects on the platform), to minimize the possibility of wrong or invalid allegations. The list was then refined into our list of 27 confirmed scams and 75 highly suspicious campaigns, thus 102 in total, based on the following criteria: (i) No promised deliveries were made to the backers after the expected delivery date (though admittedly there still might be a chance that someone might have received the product but never left a comment for 16 months after the estimated delivery date, at any place we looked for), (ii) there are no signs in any places including the Comments section and public forums, that the allegation has been resolved. When a given project meets the criteria (i) and (ii), it is included in our list of (highly) suspected scams. (iii) Furthermore, if a campaign has also received immense criticism as a fraud through press media coverage e.g., Forbes.com, CNNMoney.com etc., it is labeled as a well-known fraudulent case. We also collected data of 150 Non-Scam campaigns from successfully delivered projects, based on their contents in Updates and Comments. We admit that our dataset is still research grade, yet it consists of a hundred cases rigorously reviewed, whose size is comparable to those used in previous research [49,50].

### 3.2. Features

This section explains four categories of features we propose to use in our experiments.

#### 3.2.1. Generic Project-Based Features

We use ten generic, project-based features including the number of backers, the total number of updates, the total number of public updates (can be viewed by anyone), the funding goal (amount in $ project creator wants to raise for the project to be successful), the pledged amount (amount in $ raised during funding period), the number of comments by creator, the number of comments by backers, presence of introductory video, and the number of backers who pledged to a project to seek the rewards. Besides, we also consider the number of videos and images in Campaign and Updates sections.

#### 3.2.2. Project Creator’s Features

We use some features from the creator’s profile showing their cordiality, i.e., features depicting their social traits such as existence of a link to a Facebook ID, the number of external links to websites, the number of the project creator’s comments left on other projects, etc. We also consider features related to the creators’ prior experiences on the platform, such as the number of created projects, the number of backed projects, and time (in hours) elapsed from the creation of his/her account until they launch the project.

#### 3.2.3. Linguistic Features

Previous research [39,51] has shown that linguistic cues play an important role in detecting lies, deception or hidden intentions of a person, which motivated us to include a set of linguistic features (shown in Table 1) in our scam detection experiments. All the features listed in Table 1 have been calculated from and then applied to each of the three sections, Campaigns, Updates, and Comments, separately.

#### 3.2.4. Named Entity Recognition (NER)

We use Stanford NER [14] to recognize three types of named entities which are person (e.g., Frank, Richard, Tony), location (e.g., Canada, UK, America), and organization (e.g., Google, Philips, Apple).

### 3.3. Performance Metrics

To measure the performance of scam classifiers we adopt four metrics: (overall) accuracy, AUC, precision, and recall.

- (Overall) accuracy: the ratio of the projects correctly classified as scams or non-scams to the total number of all projects contained in our dataset. We apply this metric to measure the accuracy of a classifier on our whole dataset.
- AUC: AUC is the area under the Receiver Operating Characteristic (ROC) curve. ROC is a probability curve that shows the True Positive Ratio (TPR) against False Positive Ratio (FPR) at various threshold values and the performance of a classification model. AUC ranges in value from 0 to 1. If the model’s prediction accuracy is 100%, the AUC score is 1.

The following two metrics are to evaluate the quality of classification results, particularly in identifying scam projects.

- Precision: the ratio of True Positives over the sum of True Positives and False Positives or the percentage of campaigns that are properly attributed to a given class (scam). True Positives are the number of correctly classified scams, False Positives are the number of non-scam projects falsely ascribed to scam, and False Negatives are the number of scam projects that are falsely labeled as non-scam.
- Recall: the ratio of True Positives over the sum of True Positives and False Negatives or the percentage of scam projects (in our dataset) that are correctly identified.

## 4. Results

In this section, we first focus on extracting discriminative and informative features for identifying scam projects. Once we obtain the key features of scam campaigns, we explore the feasibility of building an accurate and early classification model for scam detection.

### 4.1. Distinguishing Characteristics of Scams

Feature selection is an important task in selecting a subset of suitable features to construct a model, particularly in the case of classification. A Logistic Regression model can be used to predict the probabilities of the classes on the basis of input features, after classifying them according to their prediction model [52]. In our experiments, a Logistic Regression model is applied for both feature selection and classification of scam and non-scam projects, as follows. As we have a large set of 157 features, we propose to use the feature selection method called Forward Stepwise Logistic Regression [15,53,54]; Starting with a simple model with no features, the algorithm progressively adds more features and assesses the performance of these features. Hence, in each step, only features determined to be significant by the Logistic Regression algorithm are added to the model. As for the question of whether a project is a scam or not, we modeled it as a binary dependent variable, with scam projects having a value of 1 and non-scams a value of 0. As a result, we reduce the size of the feature space from 157 to 17, obtaining 17 key distinguishing features of scams as shown in Table 2.

Six, four, five, and two features are selected from the Comments section, creator-based information, Updates section, and Campaign section, respectively. Generic project-based features, except for the number of images included in Campaign and Updates, were not of much help in detecting scams. Table 3 shows the results of classification performance when each category of features was exclusively used to build a classification model using Logistic Regression. We also performed Variance Inflation Factors (VIF) to check the multicollinearity of features and found no strong correlation. Overall, features extracted from the Comments section and creator-related information are found to be good indicators for scam detection, followed by the Updates section and Campaign section, achieving 72.6%, 71.4%, and 69.8%, and 60.7% accuracy, respectively. Our model achieves up to 87.3% accuracy when built with all the 17 selected features and the Logistic Regression algorithm.

#### 4.1.1. Creator-Related Features

**Figure 3:**
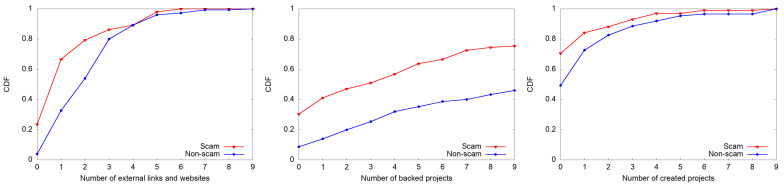
Cumulative distribution function(CDF) of key creator-related features.

Creator-related features such as whether there is a link to a Facebook ID, the number of external links and websites, the number of the creator’s backed projects, and the number of created projects are found to be significant features in our model. We found that having a Facebook ID with (β = −1.326, *p* < 0.01) and external links with (β = −0.665, *p* < 0.001) reduces the probability of being a scam by 74% and 49%, respectively. We observed that only 35% of scam projects have a link to a Facebook ID, whereas 55% for non-scams have such links. Similarly, on average 1.57 and 2.51 external links were found in scam and non-scam campaigns, respectively. Figure 3 shows the cumulative distribution function (CDF) of the selected key creator-related features. As shown in Figure 3a, 66.7% of scams (i.e., twice as many as non-scams) have 0 (23.5%) or 1 (43.1%) an external link (to websites), whereas only 4% and 28.6% of non-scam projects have 0 or 1 external link. These results indicate that scammers are, in comparison with non-scammers, more reluctant to reveal or provide their own personal or additional information.

We also found that non-scammers tend to be more actively engaged in investing activities on the crowdfunding platform than scammers. As shown in Figure 3b, 30.3% of scammers have not backed any other projects, which is 3.5 times more than non-scammers. It has been known that creators with more backing history often indicate successful fundraising as well; campaigns started by creators who have previously invested in other campaigns tend to attract more backers and collect more funds [23]. As shown in Figure 3c, 70.5% of scammers have not launched any other projects. According to our analysis, 24.5% of scammers have neither backed other projects nor created their own projects before. Overall, our results show that creators having more experience on the platform in terms of backing and launching projects are less likely to set up a scam campaign.

#### 4.1.2. Features from Campaign Section

Our model with two features selected from the Campaign Section (as shown in Table 2), i.e., redundancy and the number of images included, achieves 60.7% classification accuracy. Notably, scams tend to contain more images in their Campaign section; on average scams contain 17.1 images in a Campaign, whereas non-scams have 13.5 ones. It has been known that information contained in the Campaign section is less predictive of the success of crowdfunding campaigns as well [20,27], whereas those extracted from the Updates and Comments sections often serve as good predictors of success [20,22], which is in line with our results.

#### 4.1.3. Features from Updates Section

Out of all the selected 17 features, the Updates and Comments section contain 5 and 6, respectively, for which our model achieves 69.8% and 72.6% classification accuracy, respectively.

According to our analysis, the more often third person pronouns are used in the Updates section (β = 0.285, *p* < 0.01), the more it is likely to be a scam project, which is consistent with previous literature; liars have been found to use more third person pronouns than truth-tellers, as a way of distancing themselves and avoiding ownership of the deceptive stories [55].

Non-scam projects contain more than twice as many location names (e.g., Hong Kong, New York, etc.) as scams. Deceivers try to hide and avoid mentioning or disclosing their information like spatial information due to the deceiver’s dilemma [56,57]; liars are reluctant to mention verifiable details and they tend to provide unverifiable details instead. Particularly, they find it difficult to tell lies with spatial information, because they additionally have to create fake, imaginative writing when trying to describe a space or place they have not experienced [58,59].

Presence of an email address in the Updates section comes out to be one of the best predictors of non-scams in our model (β = −4.551, *p* < 0.05). This result also can be interpreted as a hesitation by scammers to publicly share their direct contact information, along with our results on the lower availability of the creator’s Facebook ID and external links in scams.

Notably in Updates, contrary to the results obtained in the Campaign section, we found that scammers put fewer images in the Updates section compared to non-scammers (shown in Table 2), which indicates that scammers find it more difficult to share fake updates in image data format (than text). We also found that non-scammers use past tense verbs relatively more than scammers (β = −0.686, *p* < 0.05), as they typically have more actual things to update, and those updates mostly refer to real things that happened or work done so far, thus creators often naturally tend to write in the past tense when it comes to reporting or describing them.

#### 4.1.4. Features from Comments Section

Compared to the other sections, the Comments section contained the best features for accurate scam detection, with which our model achieved the highest classification accuracy (72.6%) and AUC (0.80).

It has been known that liars use first person pronouns at a lower rate than truth-tellers [51,55]. Using first person pronouns indicate that they are being honest with themselves by subtly proclaiming ownership of a statement, whereas liars attempt to disassociate themselves from the lies by choosing to project less of themselves in their words, as they do not contain one’s true attitudes or experiences [51,55]. These observations are consistent with our results in the Comments section. We found that the more first person plural pronouns are used (β = −1.07, *p* < 0.001), the less it was likely to be a scam.

We also found in the Comments section that the more third-person pronouns are used (β = −1.971, *p* < 0.001), the less it is likely to be a scam, which, interestingly, is inconsistent with our own results obtained from the Updates section above. It is possible that this reflects the fact that the content in the Comments section mostly consists of interactive communications between creators and backers, and truth tellers interact with backers more heavily than scammers in it, as suggested by another finding of ours; The increasing number of sentences per comment (by creators) decreases the probability of being a scam by 42% (β = −0.539, *p* < 0.05), which shows that non-scammers are more active (or have more things to say at least) in responding to their backers, during which they use both second-person and third-person pronouns at a higher rate than scammers, referring to those who they are interacting with.

On the contrary, scammers use verbs at a higher rate than non-scammers, which is consistent with previous literature [43,44,57]. According to our prediction model, the use of verbs per comment increases the probability of being a scam by 130% (β = 0.835, *p* < 0.001). On average, scammers used 13.9 verbs per comment, while non-scammers used 9.9 verbs. Particularly, scammers turned out to use modal verbs at a higher rate than truth-tellers. The use of modal verbs with uncertainty such as ability, permission, probability, and obligation (e.g., can, could, may, might, will) mainly indicates uncertain facts or predictions and avoids fact-based conversations that give investors solid confidence [43,44]. Table 4 shows examples of scammers’ comments using modal verbs, found in our dataset. Finally, We observed more usage of present tense verbs in the comments of scammers than non-scammers, which is also consistent with previous literature [57].

### 4.2. Detecting Scams: Performance Evaluation

In this subsection, given the 17 key discriminating features of scam campaigns, we first evaluate the classification performance of six commonly used machine learning algorithms and then explore the feasibility of building an early scam detection model.

To build and test scam classification models, we leverage six often used machine learning algorithms: Logistic Regression, Random Forest, Support Vector Machine (SVM), Naive Bayes, k-Nearest Neighbor (KNN), and J48 Decision Tree. For the evaluation, we applied 10-fold cross validation, where we use 90% of the dataset as training data and the remaining 10% as test data. According to the results presented in Table 5, Logistic Regression turns out to be the best algorithm for accurate scam classification (with 87.3% accuracy, 0.939 AUC, and 84.3% of scam precision and recall), followed by Random Forest (79.0% accuracy and 0.851 AUC), SVM (73.4% accuracy and 0.719 AUC), Naive Bayes (70.6% accuracy), k-Nearest Neighbor (69.8% accuracy with k = 9), and J48 Decision Trees (66.3% accuracy).

We next explore the feasibility of early scam detection using our proposed key features and best-performed Logistic Regression model. Previous research [60] showed that most backers invest in the first and last weeks of a project, and the best strategy is to procure investment from backers at the beginning of the project [61]. Needless to say, it is more desirable to detect scams at an early stage of the project campaigns to minimize fraud risk, particularly before collected money is transferred to the creators after funding has ended.

**Figure 4:**
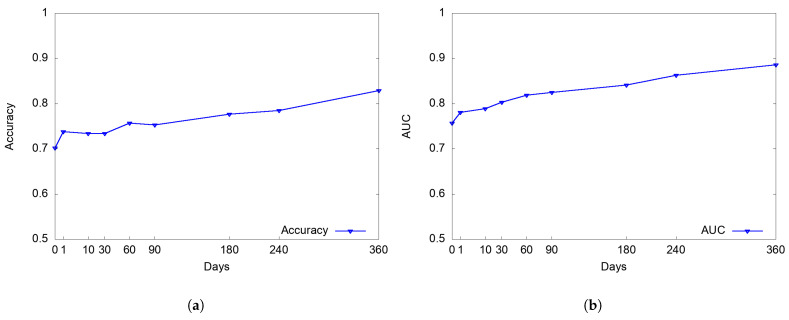

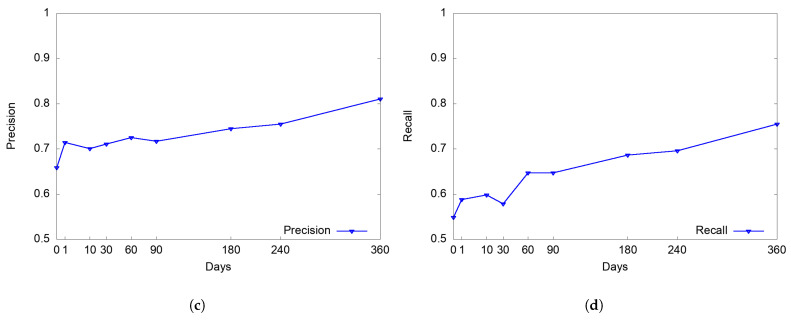
Estimated Average Classification Performance vs. Elapsed time (days). (**a**) Accuracy. (**b**) AUC. (**c**) Precision on Scams. (**d**) Recall on Scams.

Figure 4 plots the average classification performance over elapsed time after project launch. On the very first day (i.e., 0-day) of project creation, (as shown in Table 2) with only six features available by then in the Campaign section and creators’ profile and behavior information such as a link to a Facebook ID, the number of external links & websites, backed or created projects, redundancy and the number of images in a Campaign, our model achieves 70.2% accuracy (with 0.757 AUC) in detecting scams. We found that the number of created projects beforehand was a good precursor particularly for early identification of non-scam projects; our model achieves 59.5% accuracy (with 0.606 AUC) when used only with the feature. Our results indicate that the Campaign section and creator-related information contain useful information for early scam detection, yet the accuracy increases further as time goes by, with more information becoming available from Updates and Comments; the accuracy increases from 70.2% to 73.8% in just one day after project launch. Within one or two months, which is often the recommended or maximum length of crowdfunding campaigns on Kickstarter, respectively, our model’s performance increases to 73.4–75.7% accuracy, and then up to 82.9% in one year.

As shown in Figure 4c,d, during the initial two months of the project (i.e., the maximum funding period), recall increases from 54.9 to 64.7%, whereas precision reaches 65.9 to 72.5%, which is relatively (7.8–11%) higher than recall. Then, precision increases to 71.7%, 74.4%, and 81.1%, while recall increases to 64.7%, 68.6%, and 75.5%, respectively, in 3, 6, and 12 months. This indicates that as time goes by, as more data from the Updates and Comments sections arrive, our scam detection model becomes more precise as well as complete.

## 5. Conclusions

Despite a growing concern over the increased threat of fraudulent crowdfunding campaigns, little is known about them mainly due to the lack of measurement data collected from real scam cases. In this paper, we collected and analyzed a hundred crowdfunding scam campaigns using a wide variety of traits and feature selection processes. We found 17 key features of scams, six of which were our original findings, and then discussed our findings and insights on distinguishing characteristics of those fraudulent projects. Based on our findings, We built a scam detection model with 87.3% accuracy. We also explored the feasibility of early scam detection, discussing what features are more helpful, particularly at the time of project launch and thereafter.

Our work has a limitation. There is no legal proof or evidence that our scam dataset consists of 100% absolute frauds. We admit that our dataset is still research grade, yet it consists of a hundred fraudulent cases rigorously reviewed, whose size is comparable to those used in previous research.

## Figures and Tables

**Table 1:** Linguistic cues and their descriptions.

Quantity
1. (Total # of) words, adverbs, clauses, verbs, phrases, characters, punctuation, nouns, sentences, adjectives, noun phrases
(a phrase consisting of a noun, its modifiers and determinants)
**Complexity**
**2. Average # of clauses**: total # of clauses/total # of sentences
**3. Average sentence length:** total # of words/total # of sentences
**4. Average word length:** total # of characters/total # of words
**5. Pausality:** total # of punctuation marks/total # of sentences
**Non-immediacy**
**6. Self reference:** total # of first person singular pronouns
**7. Group reference:** total # of first person plural pronouns
**Uncertainty**
**8. Modal verbs:** a verb that is usually used with another verb to express ideas such as possibility, necessity, and permission
**9. Other reference:** total # of second and third person pronouns
**Expressiveness**
**10. Emotiveness:** total # of adjectives + total # of adverbs/total # of nouns + total # of verbs
**Diversity**
**11. Lexical diversity:** percentage of unique words (total # of different words/total # of words)
**Redundancy**
**12. Redundancy:** total # of function words/total # of sentences
**Informality**
**13. Typo ratio:** total # of misspelled words/total # of words
**Relativity**
**14. Time:** total # of time, e.g., hour, o’clock, evening, yesterday etc.
**15. Past, present and future tense verbs:** total # of past, present and future tense verbs

**Table 2:** Key features selected by Logistic Regression for scam detection and their descriptive statistics. Nagelkerke R^2^ = 0.706. Significance: *** *p* < 0.001, ** *p* < 0.01, * *p* < 0.05, β is the measured coefficient for each feature in the model’s equation. If *p-value* is less than 0.05 then it becomes more significant for the model.

					Scam		Non-Scam	
		β	SE	*p*-Value	Mean	SD	Mean	SD
**Creator**	Existence of a link to a Facebook ID	−1.326	0.446	**	0.350	0.480	0.550	0.499
	Num. external links & websites	−0.665	0.169	***	1.570	1.570	2.510	1.570
	Num. backed projects	−0.042	0.015	**	8.740	15.327	22.550	34.270
	Num. created projects	−0.320	0.150	*	1.730	1.536	2.380	2.656
**Campaign**	Redundancy	0.206	0.128	0.108	5.367	2.819	4.887	1.699
	Num. images	0.060	0.021	**	17.090	16.690	13.470	11.226
**Updates**	Num. third person pronouns/Num. updates	0.285	0.101	**	3.653	2.943	4.353	5.091
	Num. images/Num. updates	−0.488	0.222	**	0.777	1.087	1.017	1.248
	Num. emails/Num. updates	−4.551	1.978	*	0.046	0.095	0.159	0.260
	Num. location/Num. updates	−1.585	0.402	***	0.544	0.670	1.086	1.286
	Num. past tense verbs/Total words	−0.686	0.272	*	0.025	0.010	0.028	0.007
**Comments**	Num. verbs/Num. creator comments	0.835	0.140	***	13.906	10.562	9.916	5.407
	Num. sentences/Num. creator comments	−0.539	0.214	*	3.819	2.621	3.374	1.944
	Num. first person plural pronouns/Num. creator comments	−1.070	0.276	***	1.799	1.791	1.726	1.179
	Num. second person pronouns/Num. creator comments	−1.068	0.339	**	1.756	1.561	1.660	1.014
	Num. third person pronouns/Num. creator comments	−1.971	0.542	***	1.310	1.056	1.071	0.831
	Num. present tense verbs/Total words	0.151	0.076	*	0.119	0.028	0.115	0.024

**Table 3:** Performance of our model built with each category of features using Logistic Regression (Precision and Recall on Scams).

Feature	Precision	Recall	Accuracy	AUC
Creator-related	65.3%	62.7%	71.4%	0.758
Campaign	55.5%	14.7%	60.7%	0.593
Updates	62.5%	**63.7%**	69.8%	0.752
Comments	**70.3%**	55.8%	**72.6%**	**0.805**
Full model	84.3%	84.3%	87.3%	0.939

**Table 4:** Scammers’ comments using modal verbs: examples.

*“We **might** be a couple days behind schedule”*
*“We **could** make it happen faster, but as we are having the game printed in china, it will take some time to get them literally shipped overseas after they are produced.”*
*“We know it is a bummer you **will** not be able to play it on your computer right away, but we **will** still have it out for you by september 2013”*
*“I know we **can** do it”*
*“I **can** only tell you that i **will** use my best endeavors to make it happen.”*

**Table 5:** Performance comparisons of different classification algorithms (10-fold cross validation).

Algorithm	Precision	Recall	Accuracy	AUC
Logistic regression	**84.3%**	**84.3%**	**87.3%**	**0.939**
Random Forest	77.5%	67.6%	79.0%	0.851
SVM	68.4%	63.7%	73.4%	0.719
Naive bayes	61.8%	71.5%	70.6%	0.734
KNN (k = 9)	66.6%	50.9%	69.8%	0.757
J48 Decision Tree	58.4%	57.8%	66.3%	0.660

## Data Availability

Not applicable.

## Abbreviations

The following abbreviations are used in this manuscript:	
FinCEN	Financial Crimes Enforcement Network
FTC	Federal Trade Commission
VC	Venture Capital
NER	Named Entity Recognition
ROC	Receiver Operating Characteristic
AUC	Area Under the Receiver Operating Characteristic
TPR	True Positive Ratio
FPR	False Positive Ratio
CDF	Cumulative Distribution Function
SVM	Support Vector Machine
KNN	k-Nearest Neighbor
VIF	Variance Inflation Factors
